# Perceived Stress and Outness: Examining the Coping Mediation Framework Among Chinese LGBTQ+ Community

**DOI:** 10.3390/bs14110978

**Published:** 2024-10-22

**Authors:** Chenwei Ma, Jiangyu Li

**Affiliations:** School of Public Administration, Sichuan University, Chengdu 610065, China; cxm782@163.com

**Keywords:** perceived stress, proactive coping, preventive coping, outness, Chinese LGBTQ+, mental health

## Abstract

Despite global progress in LGBTQ+ rights, sexual minorities in China face significant societal pressures and legal ambiguities, which remain poorly understood. This study explores the potential mediating role of proactive and preventive coping strategies in the relationship between perceived stress and outness levels among Chinese LGBTQ+ individuals. Survey data from 267 Chinese LGBTQ+ individuals aged 16–42 revealed high levels of perceived stress within this community. Both proactive and preventive coping strategies significantly mediated the negative impact of perceived stress on outness levels. These findings contribute to the understanding of LGBTQ+ community’s perceived stress and outness status in China, highlighting the need for inclusive policies and support systems to improve their mental health and social well-being.

## 1. Introduction

Despite progress in some regions, the global LGBTQ+ community still faces significant discrimination and systemic injustice, particularly where legal protections are limited [1,2]. In China, although homosexuality was declassified as a mental illness by the Chinese Society of Psychiatry in 2001, the LGBTQ+ community continues to struggle within a legal gray area [3]. Moreover, the emphasis on family dignity and moral righteousness in Chinese society further complicate the situation for the LGBTQ+ community [4]. Research indicates that LGBTQ+ individuals in China face significant discrimination and marginalization across various social contexts and often resort to concealing their sexual identities as a coping mechanism [5]. When it comes to hiding their sexual identities, people weigh the risks and benefits of being honest and open with one another in interpersonal communication [6]. If the perceived risk is greater than the potential reward, people may choose to use disclosure avoidance as a protective tactic rather than taking the initiative to come out as themselves. Most notably, an earlier study indicates that a person’s propensity to reveal is triggered by perceived lower levels of stress [7].

While the growing global literature on LGBTQ+ identity, perceived stress, and mental health emphasizes the importance of future-oriented coping strategies within the community [8,9,10], studies specifically examining this aspect among LGBTQ+ individuals in China remain limited. Existing studies primarily focus on the gay community and their stress, challenges, and lives [4,11,12]. Addressing these gaps, the present study examines the extent of outness among Chinese LGBTQ+ individuals in response to perceived pressures and investigates the impact of proactive and preventive coping strategies on this decision. Finally, this study also investigated the perceived stress and outness of LGBTQ+ individuals in China, in order to provide a comprehensive understanding of the current situation of stress and coming out of the LGBTQ+ individuals in China and to enrich the existing research.

## 2. Perceived Stress of LGBTQ+ Community in Chinese Society

The Chinese LGBTQ+ community faces significant pressures, including social rejection, family pressure, and workplace discrimination, causing mental health issues [5,13]. Despite some progress over the past few decades, attitudes towards LGBTQ+ individuals in China remain complex and contradictory. While Chinese law does not explicitly prohibit homosexual behavior, there are also no anti-discrimination laws or legal recognition of same-sex marriage [5]. Consequently, homosexual behavior is treated like the elephant in the room in mass media, social opinion, and public education—it exists but cannot be acknowledged [14].

Hjarvard (2008) [15] explains how societal norms and behaviors are shaped globally through news, social, and online platforms, contributing to more tolerant views on homosexuality. However, in China, where homosexuality is considered a sensitive topic, societal attitudes remain cautious and conservative, reflecting the official stance [14]. The State Administration of Press, Publication, Radio, Film, and Television (SAPPRFT) employs a strict censorship process for TV shows and films, from script approval to final production. Although official documents like “Regulations on TV Drama Content Management (2010)” and “Film Industry Promotion Law (2016)” do not explicitly ban homosexual themes, vague and non-transparent review standards have led to specific guidelines. In 2016 to 2017, the media industry associations created the “General Rules for TV Drama Content Production (2016)” and “General Rules for the Review of Online Audiovisual Program Content (2017)”, which explicitly state that “depictions of abnormal sexual relationships or behaviors, such as incest, homosexuality, sexual perversion, sexual assault, sexual abuse, and sexual violence”, are considered “exaggerations of pornographic and vulgar interests” and must be “deleted or modified”.

Before these guidelines, the increasing exposure of LGBTQ+ themes in film, television, and online platforms in China has gradually fostered a greater societal understanding and acceptance of the LGBTQ+ community [16]. However, the enactment of these rules has resulted in the nationwide ban and removal of films and TV shows with homosexual content. Even airing shows with LGBTQ+ themes, such as “Go Princess Go” (2015) and “Addicted” (2016), were cut off or re-edited and removed from all platforms mid-broadcast [17]. This regulatory environment reflects the broader societal challenges faced by the LGBTQ+ community in China, where homosexuality is still often regarded as taboo, and public acceptance remains limited [18].

In the absence of official discourse, the public, especially the older generation, habitually judges the LGBTQ+ community based on traditional social and moral standards [5]. The emphasis on family dignity and moral ethics in Chinese traditional culture places significant pressure on the LGBTQ+ community [19]. Deeply ingrained notions of family lineage and procreation lead many LGBTQ+ individuals to hide their sexual orientation to avoid family conflict and social rejection [4]. This pressure became particularly evident during the pandemic, when many LGBTQ+ individuals had to spend extended periods with their family members, facing increased scrutiny and discrimination [20].

Sexual minorities still face significant discrimination and marginalization in the workplace. Research by Wang et al. (2020) [5] indicates that LGBTQ+ individuals in China encounter considerable discrimination during job searches and career development, leading many to conceal their sexual orientation to avoid unfair treatment. This concealment not only impacts their career development but also has negative effects on their mental health.

### 2.1. Future-Oriented Coping and Coming Out

A future-oriented coping theory involves proactive coping and preventive coping strategies aimed at managing potential future stressors [1]. Proactive coping involves building resources to pursue challenging goals and foster personal growth, whereas preventive coping focuses on developing resistance resources to reduce the severity of stress and the likelihood of future stressful events [21,22]. For the LGBTQ+ community, especially in conservative societies like China, the complexities of coming out and managing societal pressures necessitate the use of these strategies [23]. Although direct research on this topic is limited, numerous studies on the social pressure, marital, and living conditions of the Chinese LGBTQ+ community demonstrate the use of both proactive and preventive coping strategies to manage stress.

### 2.2. Proactive Coping Strategy

Studies show that the LGBTQ+ community employs proactive coping strategies, such as building supportive social networks, engaging in rights movements, and accessing mental health resources, to effectively manage stress and challenges [10,24]. Psychological well-being among Chinese LGBTQ+ individuals positively correlate with parental respect and perceived support for their sexual orientation across age groups [25]. Wang (2020) [5] further argues that successful “out” lesbians in urban, middle-class Chinese households tend to experience improved communication and tolerance with their mothers. Additionally, Chinese LGBTQ+ individuals often rely on online peer support within their community to mitigate friend discrimination and avoid rejection [26]. Despite these coping mechanisms, the LGBTQ+ community in China experiences limited social support from family and friends compared to Western contexts [5,27].

### 2.3. Preventive Coping Strategy

Minority stress model [8] posits that the LGBTQ+ community experiences increased stress due to prejudice, discrimination, and the need to conceal their identity, resulting in significant mental health issues. To cope with these stresses, Chinese LGBTQ+ individuals employ preventive strategies such as entering “fake” or cooperative marriages between lesbians and gay men to meet societal and familial expectations while maintaining same-sex relationships [4]. Others engage in mixed-orientation marriages, hiding their same-sex attraction and marrying unsuspecting heterosexual partners [8]. Research shows that only about 5% of sexual minority individuals in China come out in settings such as schools and workplaces. Among sexual minorities, 84.1% are married to heterosexual partners, 13.2% are in cooperative marriages, and only 2.6% have registered same-sex marriages abroad [28]. In addition, Shi et al. (2020) [25] discovered that Chinese gay and bisexual men who perceive greater family support may experience increased pressure to marry and have children due to family-oriented cultural expectations.

### 2.4. Coming Out (Outness)

Coming out is identified as a positive coping strategy that improves mental health, self-esteem, and job satisfaction while reducing perceived discrimination [7,29]. Especially for those with social support, coming out fosters authentic self-expression and reduces internal conflict and stress [30]. Identity concealment may avoid direct discrimination and social exclusion [8], but it often leads to significant long-term stress and mental health issues.

Whilst some scholars hold different views, Pachankis et al. (2015) [31] found that the mental health effects of sexual orientation concealment or disclosure differ by gender. They argue that closeted men are less likely to be depressed than out men, while out women have lower odds of depression compared to closeted women, highlighting the need for gender-specific clinical and policy approaches. Devito et al. (2018) [32] discovered that social media users in the US tend to utilize their “personal social media ecosystems” to avoid stigma while expressing their identity and adjusting their presentation strategies on outness over time. Similarly, most Chinese LGBTQ+ individuals carefully plan the timing and context of coming out to minimize discrimination and social isolation [14]. Bie and Tang (2016) [19] analyzed 60 online self-reported stories from Chinese gay men and found that their coming-out strategies are influenced by relationships with parents, peers, and wives, and by social expectations regarding the dynamics between a son and his parents, a man and his peers, and a husband and his wife.

## 3. The Present Study

According to the literature review, perceived pressures continuously impact the lifestyle and mental health of LGBTQ+ individuals. However, research on the Chinese LGBTQ+ community’s perceived pressure and their willingness to come out is limited. Proactive coping strategies, such as making adequate preparations and coming out openly, and preventive coping strategies, such as identity concealment to avoid harm, are self-protection measures largely adopted by LGBTQ+ individuals when facing stress. Although several scholars have highlighted the importance of coping for the Chinese LGBTQ+ community, little research has examined the relationship between these strategies and the coming-out process. This study aims to explore these dynamics and their impact on individuals’ decisions to disclose their sexual orientation. By doing so, it provides further insight into the coping mechanisms of LGBTQ+ individuals and how these strategies influence their decisions to come out.

Perceived stress involves an individual’s assessment of adversity in their environment [33]. Sexual minorities, particularly the LGBTQ+ community, often experience higher stress levels due to marginalization and discrimination compared to heterosexual individuals [34]. In China, limited legal protections and Confucian values emphasizing traditional family roles exacerbate these stressors [35,36]. Outness, or openly revealing one’s sexual orientation, is influenced by such external pressures. Studies indicate that greater outness correlates with increased vulnerability to victimization, leading some individuals to conceal their identity as a protective measure [8]. Thus, perceived stress might negatively impact the level of outness among Chinese LGBTQ+ individuals.

According to Conservation of Resources (COR) Theory [37], individuals strive to protect and accumulate resources to cope with stress effectively. For Chinese LGBTQ+ individuals, proactive and preventive coping mechanisms play crucial roles in resource management, while anticipating future challenges helps to mitigate the adverse effects of stress on outness by preserving and acquiring resources needed to navigate challenging social environments [38].

Therefore, we propose three hypotheses:

**Hypothesis** **1.** *Perceived stress is negatively related to outness among Chinese LGBTQ+ individuals.*

**Hypothesis** **2.** *Proactive coping mediates the negative relationship between perceived stress and outness.*

**Hypothesis** **3.** *Preventive coping mediates the negative relationship between perceived stress and outness.*

## 4. Methods

### Participants

This study was conducted from October 2023 to March 2024 in China using a mixed approach involving snowball sampling and network survey methods. An electronic survey, including validated scales, was prepared and distributed through several LGBTQ+ online platforms and social networking apps for the LGBTQ+ community. The questionnaire included sections on personal information, scales of outness status, perceived stress, proactive coping, and preventive coping. Written informed consent was obtained from all participants before enrollment, and the questionnaire data were anonymized. Participants were informed about the survey’s target population, important details, and their right to participate voluntarily and anonymously. Submission of the completed questionnaire indicated their consent.

The inclusion criteria for the study were as follows: (1) Chinese, (2) self-identified as a member of LGBTQ+ community, and (3) 16 years of age or older. The study initially received 368 submissions, with a total of 267 valid questionnaires after screening.

## 5. Measures

### 5.1. Perceived Stress

A 10-item questionnaire of perceived stress (PSS-10) was originally developed by Cohen et al. (1983) [39] and has been widely used and validated in various community samples, including marginalized groups facing high levels of stress [34,40]. As recommended by Cohen and Williamson (1998) [39], the PSS-10 (10-item Perceived Stress Scale) was used in this study due to its superior psychometric properties compared to the PSS-14 and PSS-4 versions.

In this study, the PSS-10 was applied to assess perceived stress among Chinese LGBTQ+ individuals. Despite the unique challenges faced by this community, such as societal pressures and marginalization, the PSS-10 has proven to be a reliable and valid tool for measuring stress. This scale consists of 10 items (e.g., “How often have you been upset because of something that happened unexpectedly?”), which included four positive items (e.g., “how often have you felt that things were going your way?”). For all items, participants were asked to indicate their feelings using a 5-point Likert scale, from 1 = never to 5 = always. To calculate a total PSS score, responses to the four positively stated items (items 4, 5, 7 and 8) first need to be reversed (i.e., 1 = >5; 2 = >4; 3 = >3; 4 = >2; 5 = >1). A higher score shows a higher level of perceived stress. In this study, a Cronbach’s alpha coefficient of 0.726 was obtained.

### 5.2. Proactive Coping

The scale of proactive coping was assessed among the seven scales of the Proactive Coping Inventory (PCI), including the Proactive Coping Scale, the Reflective Coping Scale, Strategic Planning, Preventive Coping, Instrumental Support Seeking, Emotional Support Seeking, and Avoidance Coping, developed by Greenglass et al. (1999) [41] and adapted and applied to sexual minority groups by Craig et al. (2019) [24], which involved goal setting with self-regulatory goal attainment cognitions and behavior for future-oriented self-improvements in coping with anticipated stress.

This scale consists of 14 items (e.g., “I am a “take charge” person.”), which included three negative items (e.g., “I try to let things work out on their own.”). For all items, participants were asked to indicate their feelings using a 5-point Likert scale, from 1 = not at all true to 5 = completely true. To calculate a total PSS score, responses to the three negatively stated items (items 2, 9, and 14) first need to be reversed (i.e., 1 = > 5; 2 = > 4; 3 = > 3; 4 = > 2; 5 = > 1). A higher score shows a higher level of proactive coping. The Cronbach’s alpha coefficient for this scale was 0.942.

### 5.3. Preventive Coping

The scale of preventive coping was also assessed among the seven scales of the Proactive Coping Inventory (PCI): A Multidimensional Research Instrument Scale [41]. Different from the proactive coping, this scale focuses on obtaining advice, information, and feedback from people in one’s social network when dealing with stressors.

The scale is a single-dimensional structure with 10 items (e.g., “I plan for future eventualities.”), using a 5-point scoring system that ranged from 1 = not at all true to 5 = completely true. A higher score represents a stronger level of preventive coping. In the present study, the Cronbach’s alpha coefficient was 0.898.

### 5.4. Outness

The scale of outness, originated from the Outness Inventory developed by Mohr and Fassinger (2000) [42] and adapted by Poteat et al. (2021) [43], is administered to indicate the extent to which the LGBTQ+ individuals are out to others in their lives. Questions were asked in regard of different social networking groups by the stem, “For each of the following groups, how many people currently do you think know of your sexual orientation?” Five items were selected and included for this scale according to the Chinese culture and social environment that the LGBTQ+ community faced mostly: (a) family members, (b) classmates at school/coworker at work, (c) superiors (as teacher to student, manager to office worker), (d) friends, and (e) the predicted outness level in ten years. Response options were none, a few, some, most, and all (on a scale of 0 to 4). The Cronbach’s alpha coefficient for this study was 0.868.

The variables in this study strictly followed the “translation-back translation” procedure to translate all the scales originally developed in English into Chinese.

## 6. Data Analyses

The data were analyzed using IBM’s SPSS, version 17, and AMOS software, version 17. An absolute correlation coefficient greater than 0.7 among two or more predictors indicates the presence of multicollinearity [44]. The correlation coefficients between variables in this study were found to be less than 0.7, indicating no serious multicollinearity. Dummy codes were created for categorical variables: gender (1 = male, 2 = female), education (1 = Bachelor’s degree and below, 2 = Master’s Degree, 3 = Doctoral degree or above), identity (1 = lesbian, 2 = gay, 3 = bisexual, 4 = transgender, 5 = queer). Descriptive statistics presented the status of each variable. Spearman’s rho correlations were computed to examine the relationships between perceived stress, proactive coping, preventive coping, and outness. A structure-equation-modeling approach was adopted for supplementary analyses on testing the mediation mechanisms of proactive coping and preventive coping between Chinese-LGBTQ+-community-perceived stress and outness.

## 7. Results

First, we performed descriptive statistical analyses of demographic variables. The sample comprised 267 Chinese LGBTQ+ individuals, 192 identified as lesbian/gay (131 female, 61 male), 47 identified as bisexual (4 female, 43 male), and 28 identified as transgender or queer (6 female, 22 male). Ages ranged from 16 to 42 years old (M = 27.62 years, SD = 5.68). Demographic details are presented in Table 1.

In terms of reliability, the Cronbach’s α values of the four variables are all greater than 0.70, the standardized factor loadings are all greater than 0.60, the CR (composite reliability) values are all greater than 0.70, and the AVE (average variance extracted) values are all greater than 0.50 (except perceived stress). According to Fornell and Larcker (1981) [45], if the AVE is less than 0.5 but the CR is higher than 0.6, the convergent validity is still acceptable. These indicators suggest that the variables in this study have good measurement reliability, as shown in Table 2. In terms of validity, Table 3 shows the results of the confirmatory factor analysis, with which the four-factor model has the best fit (χ^2^ = 2061.914; df = 696; χ^2^/df = 2.963; CFI = 0.951; TLI = 0.916; RMSEA = 0.039; SRMR = 0.040). Together, these results indicate that the variables in this study can be effectively differentiated from each other.

In order to understand the stress and outness of the LGBTQ+ individuals in China, this study also conducted an analysis of variance (ANOVA). This study showed that there are differences in both stress and outness among different groups of LGBTQ people. The results are shown in Table 4.

Self-reported data could lead to common method variance (CMV). Harman’s one-way analysis of variance showed that the unrotated exploratory factor analysis identified five factors, accounting for 61.14% of the total variance, with the first principal component explaining 22.80%, which is below the 40% threshold [46]. Table 3 shows that the one-factor model fit was much less superior to the three-factor model, suggesting that CMV is not a significant issue in this study. The means, standard deviations, and intercorrelation matrix for all variables are presented in Table 5.

### Hypothesis Tests

Hypothesis 1 proposed that perceived stress is negatively related to outness. As shown in Table 6, perceived stress had a negative relationship with outness (B = −0.107, SE = 0.057, *p* < 0.01), supporting Hypothesis 1.

**Figure 1:**
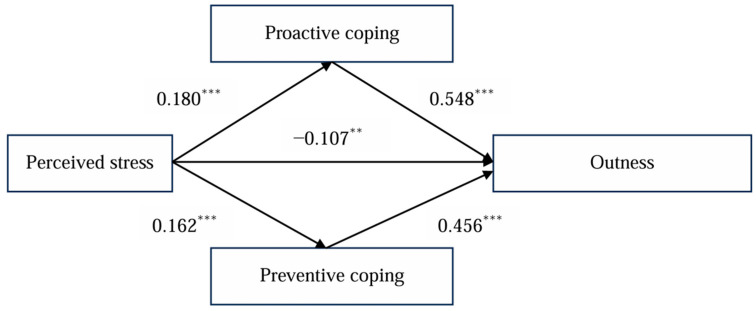
Mediation model showing the association between perceived stress and outness with proactive coping and preventive coping as mediators. Standardized regression coefficients are presented. Covariates were not presented for the sake of simplicity, ** *p* < 0.01; *** *p* < 0.001.

Previous research indicates that demographic variables such as age, gender, gender identity, and education [43] were controlled as covariates in this study. The mediation model of the relationships between two components of perceived stress and outness is presented in Figure 1.

The size of the indirect effect and its confidence interval were estimated and tested in this study using a bootstrap method. The mediation effect of proactive coping is shown in Table 7. The indirect effect of perceived stress on outness through proactive coping was significant (B = −0.098, SE = 0.032, 95% confidence interval (CI) [−0.178, −0.029]), supporting Hypothesis 2.

The mediation effect of preventive coping is also shown in Table 8. The indirect effect of perceived stress on outness through preventive coping was significant (B = −0.097, SE = 0.035, 95% confidence interval (CI) [−0.157, −0.019]), supporting Hypothesis 3.

## 8. Discussion

This study enriches the field of sexual minority groups’ future-oriented coping by examining whether and how perceived stress relates to outness within the Chinese LGBTQ+ community. Perceived stress was found to negatively correlate with outness, with proactive and preventive coping playing significant mediating roles in this relationship. These findings enhance our understanding of the positive effects and mechanisms of future-oriented coping within the Chinese LGBTQ+ community.

### 8.1. Perceived Stress and Outness in Chinese LGBTQ+ Community

This study first examined the relationship between perceived stress and outness among Chinese LGBTQ+ individuals, finding a negative correlation. Despite international acceptance reducing perceived stress for the LGBTQ+ community [36], the stress level (Mean = 3.28, SD = 0.61) in China remains high. Our statistical analysis shows that among Chinese LGBTQ+ individuals, gay individuals experience the highest stress levels (Mean = 3.78, SD = 0.68), while lesbians experience the lowest (Mean = 3.04, SD = 0.48). This could be because gay individuals are the only minority group that has come out to most people (Mean = 3.47, SD = 1.09), whereas other groups’ outness levels are all below the average of 3: lesbians (Mean = 2.83, SD = 0.93), queers (Mean = 2.5043, SD = 0.86), transgender individuals (Mean = 2.48, SD = 1.21), and bisexuals (Mean = 2.39, SD = 0.88). Generally speaking, the Chinese LGBTQ+ community faces immense social pressure and tends to adopt a partially hidden lifestyle according to the outness level (Mean = 2.86, SD = 1.02). These findings support our hypothesis that outness is negatively associated with perceived stress.

However, when asked about their anticipated outness in ten years, the Chinese LGBTQ+ community showed a high willingness to come out (Mean = 3.36, SD = 1.13), with gay individuals indicating the highest level (Mean = 3.84, SD = 1.24), followed by lesbians (Mean = 3.42, SD = 0.98). This suggests that despite currently opting for partial concealment, Chinese LGBTQ+ individuals still hope to come out in the future.

### 8.2. The Mediating Effects of Proactive Coping and Preventive Coping

This study highlights the mediating effects of proactive and preventive coping between perceived stress and outness. This suggests that the two armies of active coping and proactive prevention may help to alleviate the reluctance to outness due to stress in the LGBTQ community. In the Chinese LGBTQ+ community, individuals who adopt active coping strategies may help individuals manage perceived stress, which supports the minority-stress-coping model in the LGBTQ+ community [47,48]. Proactive coping strategies, such as building strong social support networks, developing resilience skills, and pursuing equal legal protection and rights, help them anticipate and prepare for potential stressors as challenges to overcome, thus reducing the impact of societal stigma and discrimination on their mental well-being and enabling them to live with pride [49].

Meanwhile, as mentioned earlier, the Chinese LGBTQ+ community also adopts preventive coping strategies to guard against potential harm, such as rejection by family, friends, and employment threats [5]. These strategies include selective disclosure, identity concealment, and education engagement to mitigate the negative impacts of societal stigma and discrimination. This supports the claim that the preventive strategies allow individuals to shield themselves from anticipated negative outcomes, thereby reducing their perceived stress and its impact on their lives as sexual minorities [25]. Moreover, this study finds that the preventive coping strategies also mediate the relationship between perceived stress and outness. That is, preventive coping strategies like identity concealment may provide a temporary buffer against societal stigma and discrimination, reducing immediate stress and protecting individuals in the short term until they are ready to come out [50].

### 8.3. Limitations and Future Research

This study has several limitations. Firstly, the cross-sectional data preclude inferring causality; thus, future studies should adopt a longitudinal research design. In particular, although we think that the association between perceived stress and outness is positive, it is also plausible that stress is produced by outside circumstances, a possibility that will be investigated further in the future. Secondly, the self-reported data from the LGBTQ+ community may lead to inaccuracies due to recall bias or misunderstanding of questions [51]. Moreover, respondents may not have answered authentically, especially for sensitive questions in the Chinese context. Future research could incorporate more objective measures or triangulate self-reports with other data sources. Additionally, since the maximum age of the respondents in this study was 42 years, the views of the older LGBTQ+ community were not included in this study. Although this study used snowball sampling and online voluntary recruitment, the sample may not be representative of all segments of the Chinese LGBTQ+ community, such as older respondents and respondents from rural areas. Due to social stigma and fear of discrimination, participant recruitment was limited, resulting in a relatively small sample size and large standard deviation for key variables. This limitation restricts the generalizability of the findings and may not fully capture the diversity of this population. Future research should employ more robust sampling methods, such as longitudinal designs or mixed-method approaches, to include a broader range of participants from different regions and social backgrounds, thereby providing more reliable and comprehensive insights into the experiences of the Chinese LGBTQ+ community. Finally, due to limited time and resources, this study primarily focused on the relationship between perceived stress, proactive coping, and preventive coping. Future research could examine other psychological constructs, such as resilience, internalized homophobia, and social support, or use qualitative methods, such as interviews and focus groups, to gain deeper insights into the lived experiences of the Chinese LGBT+ community and explore the nuances of their coping strategies. By addressing these limitations, future research can provide a more comprehensive understanding of the factors influencing outness and well-being in the LGBTQ+ community in China and beyond.

## 9. Conclusions

This study is the first to substantiate the mediating role of proactive and preventive coping in the relationship between perceived stress and outness within the LGBTQ+ community in mainland China. Through observations and surveys, we found that most LGBTQ+ individuals in China face significant stress, which negatively impacts their outness levels. Proactive and preventive coping strategies were identified as key individual factors, revealing a masking effect between perceived stress and coming out. By highlighting the complex interplay between stress, coping mechanisms, and outness, this study offers valuable insights into the lived experiences of the Chinese LGBTQ+ community. To reduce stereotyping, stigmatization, and discrimination, it is crucial for Chinese society to adopt a more inclusive and diverse attitude. Decreasing sexual minority-related psychological distress and stress should improve public health and overall well-being.

## Figures and Tables

**Table 1:** Descriptive statistics of demographic variables.

Variable	Categories	Frequency	Percentage
Sex	Male	72	27.0
	Female	195	73.0
Education	Bachelor’s degree and below	115	43.1
	Master’s Degree	134	50.2
	Doctoral degree or above	18	6.7
Self-identity	Lesbian	131	49.1
	Gay	61	22.8
	Bisexual	47	17.6
	Transgender	5	1.9
	Queer ^a^	23	8.6
Career	Civil servants, career staff	24	9.0
	Teachers, doctors, lawyers	40	15.0
	Enterprise employees	98	36.7
	Businessmen, employers	4	1.5
	Free-lancer	35	13.1
	Others	66	24.7

^a^ Queer: pansexual, asexual, and/or if they do not feel that the terms lesbian, gay, bisexual, or trans fully represent them.

**Table 2:** Measurement model and reliability measures.

Construct	Composite Reliability	Cronbach’s Alpha	AVE
Perceived stress	0.816	0.726	0.381
Proactive coping	0.957	0.942	0.846
Preventive coping	0.907	0.898	0.611
Outness	0.865	0.868	0.565

Note. AVE = average variance extracted. All loadings are significant at *p* < 0.001.

**Table 3:** Goodness-of-fit information for the alternative factor models.

Model	Factors	χ^2^	df	χ^2^/df	CFI	TLI	RMSEA	SRMR
4-factor model	PS; PAC; PVC; O	2061.914	696	2.963	0.951	0.916	0.039	0.040
3-factor model	PS+PAC; PVC; O	2601.259	699	3.721	0.647	0.626	0.101	0.133
3-factor model	PS+PVC; PAC; O	2999.754	699	4.291	0.573	0.548	0.111	0.164
3-factor model	PS; PAC+PVC; O	3107.787	699	4.446	0.553	0.526	0.114	0.116
2-factor model	PS+PAC+PVC; O	3517.731	701	5.018	0.448	0.448	0.123	0.127
1-factor model	PS+PAC+PVC+O	4070.566	702	5.798	0.375	0.340	0.134	0.173

Note. PS = perceived stress, PAC = proactive coping; PVC = preventive coping; O = outness.

**Table 4:** Analysis of variance.

Variable	Group	Items	M	SD	F	P	LSD
Perceived stress	L (1)	131	3.388	0.555	1.996	0.095	2 > 1 > 3 > 4 > 5
	G (2)	61	3.593	0.598			
	B (3)	47	3.371	0.457			
	T (4)	5	3.314	0.648			
	Q (5)	23	3.301	0.579			
outness	L (1)	131	2.832	0.929	9.955	0.000	2 > 1 > 5 > 4 > 3
	G (2)	61	3.466	1.089			
	B (3)	47	2.387	0.875			
	T (4)	5	2.480	1.213			
	Q (5)	23	2.504	0.863			

**Table 5:** Means, standard deviations, and correlations.

	1	2	3	4	5	6	7
1. Gender ^a^	-						
2. Age	−0.09	-					
3. Education ^b^	−0.14 *	0.44 **	-				
4. Perceived stress	0.45 **	−0.10	0.20 **	-			
5. Proactive coping	−0.14 **	0.24 **	0.22 **	0.17 **	-		
6. Preventive coping	−0.09	0.09	0.05	0.19 **	0.30 **	-	
7. Outness	−0.31 **	0.34 **	0.21 **	−0.33 **	0.29 **	0.19 **	-
Mean	1.73	27.62	1.64	2.72	3.42	4.10	2.86
Standard Deviations	0.45	5.68	0.61	0.61	0.56	0.62	1.02

Note. N = 267; ^a^ Gender coded as (1 = male, 2 = female); ^b^ Education as (1 = Bachelor’s degree and below, 2 = Master’s degree, 3 = Doctoral degree or above). * *p* < 0.05; ** *p* < 0.01.

**Table 6:** Regression analysis with outness of the closet as the dependent variable.

	B	SE
Perceived stress	−0.358 ***	0.104
Gender ^a^	−0.419 **	0.141
Age	0.417 ***	0.092
Education ^b^	0.057	0.103

Note. B = Path coefficients; SE = Standardized errors; Path coefficients are unstandardized; Number of bootstrap samples are 2000; Level of confidence is 95%; ^a^ Gender coded as (1 = male, 2 = female); ^b^ Education as (1 = Bachelor’s degree and below, 2 = Master’s Degree, 3 = Doctoral degree or above). ** *p* < 0.01; *** *p* < 0.001.

**Table 7:** Summary of path-analytic results.

	Proactive Coping		Preventive Coping		OUTNESS	
	B	SE	B	SE	B	SE
Perceived stress	0.180 ***	0.054	0.162 ***	0.052	−0.107 **	0.057
Proactive coping					0.548 ***	0.107
Preventive coping					0.456 ***	0.079
Gender ^a^	−0.317 ***	0.086	−0.134	0.102	−0.270 **	0.100
Age	0.011 *	0.007	0.009	0.008	0.033 ***	0.008
Education ^b^	0.129 **	0.063	0.018	0.077	−0.026	0.071

Note. B = Path coefficients; SE = Standardized errors; Path coefficients are unstandardized; Number of bootstrap samples are 2000; Level of confidence is 95%; ^a^ Gender coded as (1 = male, 2 = female); ^b^ Education as (1 = Bachelor’s degree and below, 2 = Master’s Degree, 3 = Doctoral degree or above). * *p* < 0.05; ** *p* < 0.01; *** *p* < 0.001.

**Table 8:** Bootstrapping results for testing moderation effect.

	Perceived stress→Proactive coping→outness		
	B	SE	95% Boot CI
Indirect effect	−0.098 ***	0.032	[−0.178, −0.029]
Direct effect	−0.107 **	0.057	[−0.226, −0.015]
	Perceived stress→Preventive coping→outness		
Indirect effect	−0.097 ***	0.035	[−0.157, −0.019]
Direct effect	−0.107 **	0.057	[−0.226, −0.015]

Note. B = path coefficients; SE = standardized errors; CI that excludes zero indicates that the indirect effects are significant; path coefficients are unstandardized. ** *p* < 0.01; *** *p* < 0.001.

## Data Availability

The data that support the findings of this study are available on request from the corresponding author. The data are not publicly available due to privacy or ethical restrictions.
